# Subarachnoid anesthesia for sacrococcygeal pilonidal disease treatment: A case report

**DOI:** 10.1097/MD.0000000000040998

**Published:** 2024-12-20

**Authors:** Zhengshan Qin, Xin Zhao, Jianguo Feng, Jie Li

**Affiliations:** a Department of Anesthesiology, The Affiliated Hospital of Southwest Medical University, Luzhou, Sichuan Province, China; b Department of Anesthesiology, Anesthesiology and Critical Care Medicine Key Laboratory of Luzhou, Southwest Medical University, Luzhou, Sichuan Province, China.

**Keywords:** case report, complication prevention, perioperative management, sacrococcygeal pilonidal disease, subarachnoid anesthesia

## Abstract

**Rationale:**

Sacrococcygeal pilonidal disease (SPD) is a chronic inflammatory condition primarily affecting young males. This case report details the perioperative anesthetic management of a patient undergoing SPD surgery under subarachnoid anesthesia.

**Patient concerns:**

A 48-year-old obese male (body mass index 28 kg/m^2^) presented with recurrent sacrococcygeal swelling, pain, and purulent discharge for 2 months. Magnetic resonance imaging revealed a pilonidal sinus in the left subcutaneous sacrococcygeal region, with additional findings of degenerative vertebral changes and left paracentral disc protrusion at the fourth or fifth lumbar vertebrae.

**Diagnoses:**

SPD with abscess formation.

**Interventions:**

Following comprehensive evaluation, the patient underwent SPD excision under subarachnoid anesthesia. Lumbar puncture was performed at the third and fourth lumbar vertebrae interspace, and 2 mL of 0.6% ropivacaine was administered, achieving a sensory block up to the eighth thoracic vertebra level. The patient experienced transient respiratory difficulty during positional change from supine to prone, necessitating immediate reassessment of the block level and appropriate management. Vital signs were closely monitored intraoperatively, with meticulous postoperative follow-up.

**Outcomes:**

The surgery was completed successful with stable hemodynamics. No significant anesthesia-related complications were observed within 24 hours postoperatively.

**Lessons:**

Thorough preoperative assessment of local and systemic infection status is essential in SPD patients undergoing subarachnoid anesthesia. Intraoperative positional changes may affect the level of subarachnoid block, requiring vigilant monitoring of vital signs and respiratory function. Postoperative care should focus on potential anesthetic complications and wound care requirements. Individualized anesthetic management strategies are crucial for ensuring patient safety.

## 1. Introduction

Sacrococcygeal pilonidal disease (SPD) is a chronic inflammatory condition predominantly affecting young adult males aged 20 to 40 years. The incidence of SPD in Western countries is approximately 26 per 100,000 population, whereas it is comparatively lower in Asian countries.^[1,2]^ While the precise etiology of SPD remains controversial, it is widely accepted as an acquired condition closely associated with hair in the gluteal fold.^[3,4]^ SPD presents with a spectrum of clinical manifestations, ranging from asymptomatic pits to acute abscesses and chronic sinuses. Diagnosis is primarily based on patient history, physical examination, and imaging studies.^[5]^ Management strategies for SPD encompass both conservative nonsurgical approaches and surgical interventions, with surgical treatment remaining the preferred and effective method for both acute abscess phase and chronic SPD.^[6]^

Recent advancements in perioperative medicine have underscored the critical role of anesthesia in the overall treatment process. Optimal anesthetic management not only facilitates smooth surgical procedures but also significantly enhances postoperative recovery, reduces complication risks, and improves long-term outcomes.^[7,8]^ Subarachnoid anesthesia represents a viable anesthetic technique for surgical management of SPD.^[9]^ However, the unique anatomical location of SPD lesions necessitates careful consideration of potential challenges and risks associated with this anesthetic approach.^[10]^

This case report details the perioperative anesthetic management of a patient with SPD undergoing surgical treatment under subarachnoid anesthesia. It elucidates key considerations and potential pitfalls in anesthetic care for this patient population, thereby providing valuable insights for clinical practice.

## 2. Case report

**Figure 1.**
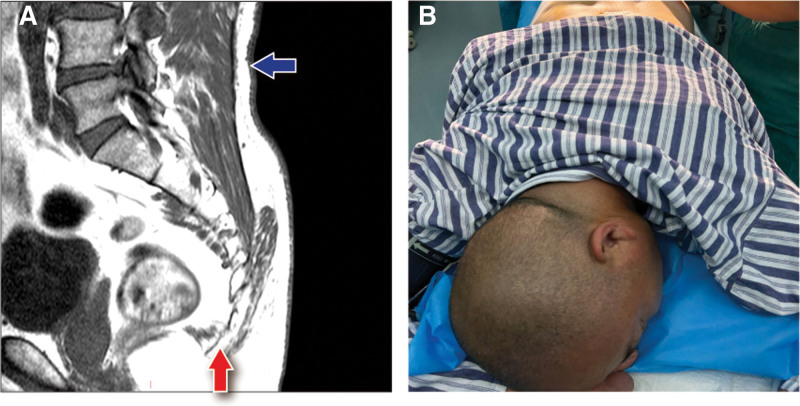
Clinical presentation and imaging of sacrococcygeal pilonidal disease. (A) MRI of the sacrococcygeal region. The red arrow points to the SPD sinus tract extending cranially. The blue arrow indicates the lumbar puncture site, demonstrating no encroachment by the SPD lesion. (B) Patient positioning diagram: transition from supine to prone position during surgical preparation that induced transient respiratory distress. MRI = magnetic resonance imaging, SPD = sacrococcygeal pilonidal disease.

A 48-year-old male patient presented with a 2-month history of recurrent pain and purulent discharge in the sacrococcygeal region. The patient’s symptoms began 2 months prior, following the consumption of spicy food. He initially experienced redness, swelling, protrusion, and throbbing pain accompanied by a burning sensation in the sacrococcygeal area. Subsequently, the lesion ruptured and discharged purulent material. Following the initial episode, the patient experienced recurrent cycles of swelling, pain, rupture, and purulent discharge in the same location. The patient’s past medical history was unremarkable, except for a positive smoking history. He was overweight, with a body mass index of 28 kg/m^2^. On physical examination, the sacrococcygeal region exhibited erythema, edema, and protrusion with marked tenderness. A ruptured sinus with purulent discharge was visible. Palpation revealed a subcutaneous cord-like structure extending towards the deep sacrococcygeal region. Magnetic resonance imaging (MRI) revealed a linear abnormal signal in the left sacrococcygeal subcutaneous tissue, suggestive of a pilonidal sinus (Fig. 1A). Additionally, degenerative changes of the spine were observed. The fourth or fifth lumbar vertebrae intervertebral disc demonstrated a left paracentral protrusion with superior migration.

Preoperative assessment included evaluation of the patient’s overall health status and presence of local infection, with exclusion of contraindications for subarachnoid anesthesia. The SPD excision was planned under subarachnoid anesthesia, with an estimated operative time of no more than 30 minutes. Upon arrival in the operating room, standard monitoring was initiated and intravenous access was established. The third and fourth lumbar vertebrae intervertebral space was selected, and lumbar puncture was performed with the patient in the lateral decubitus position. Following aseptic preparation, local infiltration was performed with 2 mL of 0.5% lidocaine. Subsequently, 2 mL of 0.6% ropivacaine was injected into the subarachnoid space with the needle bevel oriented caudally. Ten minutes postinjection, the sensory block level extended to eighth thoracic vertebra and below. During surgical preparation, as the patient was being repositioned from supine to prone, he experienced transient respiratory distress (Fig. 1B). This resolved following oxygen administration, fluid resuscitation, and positional adjustments. The operative procedure was completed in 25 minutes. Postoperatively, the patient was repositioned supine, maintained on supplemental oxygen, and monitored closely. Vital signs remained stable throughout. Follow-up on the next day revealed no significant complications such as fever, postoperative headache, nausea, vomiting, neurological dysfunction, altered consciousness, or sensory abnormalities.

## 3. Discussion

The evolution of modern anesthesiology has placed increasing emphasis on comprehensive perioperative management. The scope of anesthesia has expanded beyond intraoperative pain control to encompass comprehensive management throughout the preoperative, intraoperative, and postoperative phases, significantly influencing surgical outcomes and patient prognosis.^[11]^

Preoperative evaluation is pivotal in developing tailored anesthetic strategies. The presence of infection at the puncture site represents an absolute contraindication for subarachnoid anesthesia,^[12]^ requiring anesthesiologists to carefully assess the patient’s local and systemic infection status preoperatively. While the sinus tract in SPD typically extends cephalad, extension towards the anus can occur in rare instances, potentially leading to misdiagnosis.^[13]^ Consequently, a comprehensive preoperative evaluation serves not only to exclude infection at the subarachnoid anesthesia puncture site but also to delineate the extent of the disease. This evaluation may encompass anorectal examination, ultrasonography, and sacrococcygeal MRI.^[6]^ As demonstrated in Figure 1A, the patient’s MRI reveals that the SPD lesion does not encroach upon the potential lumbar puncture site. However, in cases presenting with extensive lesions necessitating flap reconstruction or prolonged surgical duration, general anesthesia may be the preferred anesthetic technique. This underscores the critical role of patient-specific evaluation in tailoring anesthetic management strategies.

The implications of patient positioning during intraoperative management warrant careful consideration. The patient in this case study exhibited transient breathing disorders following positional adjustment. Prone positioning during surgery is inherently associated with significant physiological alterations and a spectrum of potential complications.^[14]^ Patient positioning is a critical determinant influencing the extent of subarachnoid block.^[15]^ Alterations in patient positioning can modulate the distribution of local anesthetics within the subarachnoid space.^[16]^ Concurrently, factors such as body weight, psychological state, and restricted lower extremity mobility may contribute to patient discomfort. These observations underscore the critical necessity for vigilant monitoring of respiratory parameters and hemodynamic status during positional transitions. It is imperative to execute positional changes gradually and smoothly, ensuring the immediate availability of essential monitoring devices, resuscitation equipment, and emergency medications.

Postoperative care demands equivalent attention. Headache represents a frequent complication following subarachnoid anesthesia.^[17]^ Intraoperative positional changes invariably induce fluctuations in intraspinal pressure. Consequently, implementing a systematic follow-up protocol to evaluate complications and promptly initiate interventions is paramount. Postoperative positional management warrants careful consideration. Prolonged supine positioning may exacerbate surgical site pain. For those undergoing flap reconstruction, positional constraints may compromise flap viability. Furthermore, cases requiring negative pressure wound therapy necessitate vigilant monitoring and specialized care.

This case report emphasizes the importance of comprehensive perioperative management for SPD patients. Key elements include thorough preoperative evaluation, individualized anesthetic planning, vigilant intraoperative monitoring, and tailored postoperative care. By adopting this approach, we can enhance surgical safety and optimize patient outcomes. Future research should focus on refining these strategies to further improve clinical practice for similar cases.

## 4. Conclusion

Individualized perioperative management is crucial for SPD patients undergoing surgical treatment. Comprehensive preoperative evaluation, careful selection of anesthesia methods, vigilant intraoperative monitoring, and tailored postoperative care are essential for optimal outcomes. These insights may enhance clinical practice for similar cases.

## Author contributions

**Conceptualization:** Jianguo Feng, Jie Li.

**Data curation:** Xin Zhao.

**Funding acquisition:** Jie Li.

**Investigation:** Zhengshan Qin.

**Project administration:** Jianguo Feng.

**Resources:** Jie Li.

**Supervision:** Jianguo Feng, Jie Li.

**Writing – original draft:** Zhengshan Qin.

**Writing – review & editing:** Jianguo Feng, Jie Li.
